# “Under the hood”: artificial intelligence in personalized radiotherapy

**DOI:** 10.1093/bjro/tzae017

**Published:** 2024-07-16

**Authors:** Chiara Gianoli, Elisabetta De Bernardi, Katia Parodi

**Affiliations:** Department of Experimental Physics – Medical Physics, Faculty for Physics of the Ludwig-Maximilians-Universität München (LMU Munich), Geschwister-Scholl-Platz 1, München, 80539, Germany; School of Medicine and Surgery, Università degli Studi di Milano-Bicocca, Piazza dell'Ateneo Nuovo 1, Milano, 20126, Italy; Department of Experimental Physics – Medical Physics, Faculty for Physics of the Ludwig-Maximilians-Universität München (LMU Munich), Geschwister-Scholl-Platz 1, München, 80539, Germany

**Keywords:** radiotherapy, artificial intelligence, machine learning, deep learning, adaptive radiation therapy, personalized radiation treatment

## Abstract

This review presents and discusses the ways in which artificial intelligence (AI) tools currently intervene, or could potentially intervene in the future, to enhance the diverse tasks involved in the radiotherapy workflow. The radiotherapy framework is presented on 2 different levels for the personalization of the treatment, distinct in tasks and methodologies. The first level is the clinically well-established anatomy-based workflow, known as adaptive radiation therapy. The second level is referred to as biology-driven workflow, explored in the research literature and recently appearing in some preliminary clinical trials for personalized radiation treatments. A 2-fold role for AI is defined according to these 2 different levels. In the anatomy-based workflow, the role of AI is to streamline and improve the tasks in terms of time and variability reductions compared to conventional methodologies. The biology-driven workflow instead fully relies on AI, which introduces decision-making tools opening uncharted frontiers that were in the past deemed challenging to explore. These methodologies are referred to as radiomics and dosiomics, handling imaging and dosimetric information, or multiomics, when complemented by clinical and biological parameters (ie, biomarkers). The review explicitly highlights the methodologies that are currently incorporated into clinical practice or still in research, with the aim of presenting the AI’s growing role in personalized radiotherapy.

## Introduction

This review is structured according to the personalized radiotherapy framework as outlined in Figure 1. The definitions and descriptions of the tasks and methodologies are concisely reported in Tables 1 and 2, respectively. The aim of this review is to analyse the ways in which artificial intelligence (AI) tools currently intervene, or potentially could intervene in the future, to enhance the diverse tasks involved in the radiotherapy framework, outlined on 2 different levels: anatomy-based workflow and biology-driven workflow (Table 1). Broadly speaking, in the anatomy-based workflow AI is introduced to automate tasks and reduce time and variability with respect to conventional methodologies for the online and offline adaptation (personalization) of the treatment based on anatomical information. In the biology-driven workflow AI is conversely expected to allow for a treatment personalization guided by biological information, otherwise challenging to accomplish. Briefly explained “under the hood,” AI addresses both tasks by turning large amounts of data into models (Table 2).

**Figure:**
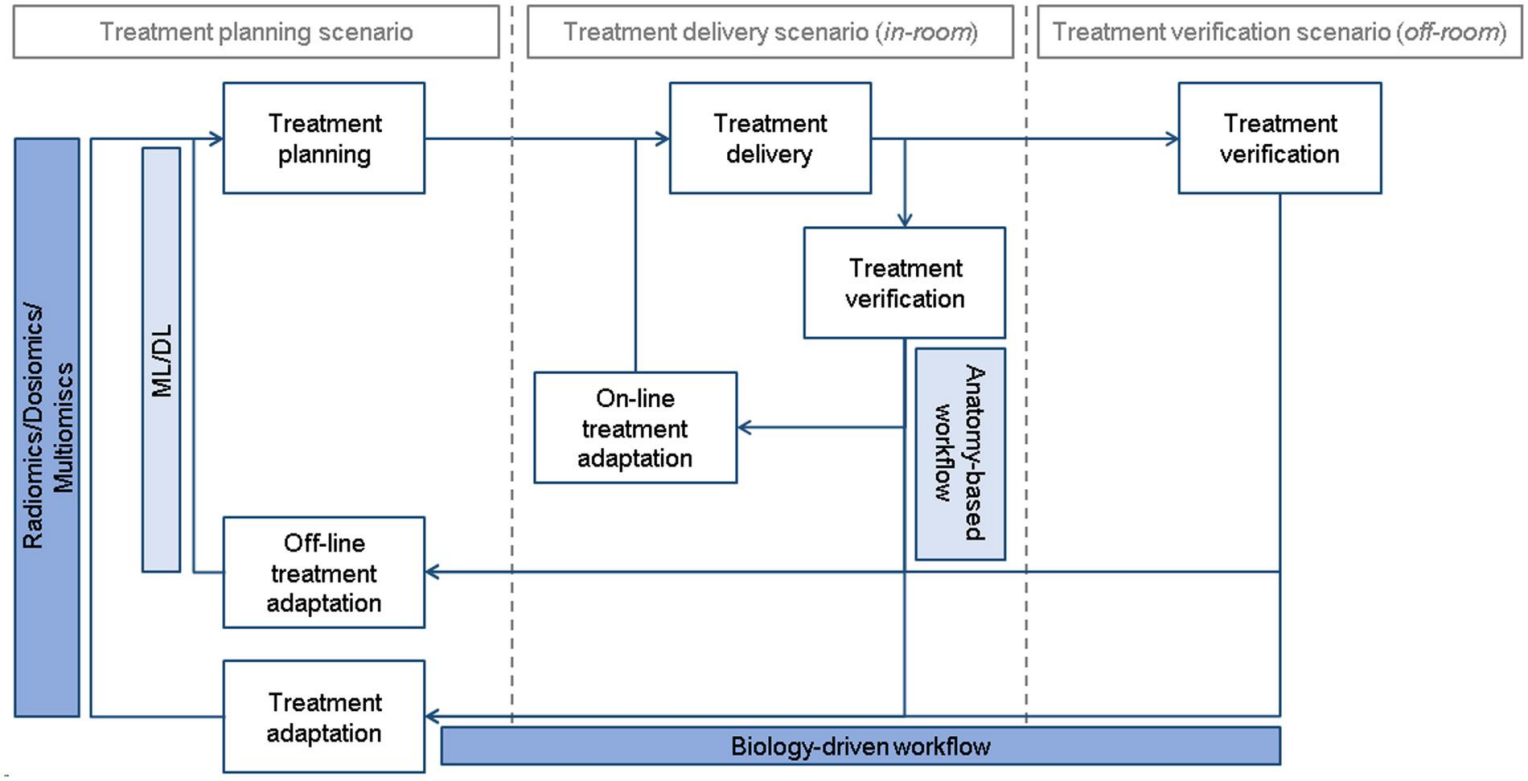
Figure 1. The anatomy-based workflow for anatomical personalization of the treatment (i.e., on-line and off-line adaptive radiation therapy, ART) and the biology-driven workflow for biological personalization of the treatment (i.e., based on biomarkers).

**Table 1.** Definitions and descriptions of the tasks involved in the radiotherapy framework covered in this review.

Definition		Description
Anatomy-based workflow	Auto-segmentation	Automatic identification of the structures in terms of tumour and the relevant organs at risk (OARs)
	Auto-planning	Automatic prediction of the dose-volume histogram or the dose distribution
		Automatic prediction of the radiation beam parameters
	Pretreatment verification	In-room anatomical imaging for the update of the patient model, prior to the treatment fraction (ie, online adaptation) or for the subsequent treatment fraction (ie, offline adaptation)
	Tumour tracking and motion compensation	Real-time, dynamic adaptation of the treatment based on time-resolved patient model and in-room imaging
	Transmission/emission-based treatment verification	Imaging of radiation induced phenomena for possible update of the patient model
Biology-driven workflow	Biology-driven dosing	Personalized dose/fraction regimen relying on outcome/toxicity prediction models and decision support systems
	Biology-driven treatment adaptation	Dose adjustment in response to changes during the fractionated course of treatment

**Table 2.** Definitions and descriptions of the AI-based methodologies relevant to this review.

Definition	Description
Artificial neural network (ANN)	Interconnected group of nodes, organized in layers, defining a model as a function of node activation functions and parameters (ie, weights and biases)
Convolutional neural network (CNN)	Interconnected groups of nodes working on structured data (ie, tensors) where the connectivity between nodes is implemented as a convolution. CNN models are trained by optimizing the convolution kernels to extract features from the tensors (ie, feature channels)
Machine learning (ML)	Methodology that builds a classification or regression model learning from examples (ie, training)
Deep learning (DL)	ML methodology based on ANNs in which multiple layers are used to extract progressively features from the data
Reinforcement learning (RL)	ML methodology in which the training is driven by interactive environment reactions as a trial-and-error learning process
U-net	Convolutional neural network made of downsampling and upsampling layers operating on the feature channels
Generative adversarial networks (GANs)	The training of generative model is framed as a supervised learning problem with 2 sub-models: the generator model that generates examples based on data and the discriminator model that tries to “judge” the generated examples. The 2 models are trained together in a zero-sum game, adversarial, until the discriminator model is fooled about half the time, meaning the generator model is generating plausible examples
Radiomics	ML methodology in which the extraction of hand-crafted or DL-derived biomarkers from medical images are used to develop predictive models
Dosiomics	ML methodology that extracts biomarkers from dose distributions of treatment plans
Multiomics	ML methodology where biomarkers are extracted also from clinical, histological and genomical data

## Treatment planning

As cornerstone of the entire radiation therapy framework, treatment planning requires the identification of the radiotherapy structures on the patient model obtained from anatomical and functional diagnostic images, the definition of the prescribed dose, and the calculation of the treatment plan to be then delivered typically in a fractionated treatment course to the patient. Commercial treatment planning systems, nowadays offering also AI-based applications, are adopted.

### Target identification

#### Anatomy-based auto-segmentation

The image segmentation of the structures in terms of tumour and relevant organs at risk (OARs) is a time-consuming process, on a slice-by-slice basis when manually performed, subject to significant inter- and intraoperator variability. Automatic segmentation (ie, auto-segmentation) enables the automation and standardization of this process.^1^ Conventional auto-segmentation is based on the image of the patient standalone, as captured by the primary X-ray CT imaging, eventually complemented with additional knowledge coming from secondary imaging such as MRI and/or PET (ie, multimodality treatment planning). Auto-segmentation based on atlas combines prior knowledge from a cohort of patients as a ground truth organ segmentation, adapted to the patient according to deformable image registration (DIR) of the anatomical images. Auto-segmentation based on deep learning (DL) instead embeds prior knowledge from the cohort of patients into a parameterized model that is optimized to match the ground truth segmentation during the training. The training is mathematically formulated as solving an optimization problem to find the model parameters that minimize a problem-specific loss function. DL enables in principle auto-segmentation of the tumour.^2^

The accuracy of the DL-based auto-segmentation is expected within the interoperator variability, as the network cannot perform better than the manual segmentation adopted as ground truth.^3^ Therefore, auto-segmentation is eventually revised and edited by clinical operators.

DL-based auto-segmentation is widely investigated in the literature, including comparison with manual segmentation^4^ as well as atlas-based auto-segmentation, and commercial DL-based software solutions for organ auto-segmentation are currently available. In general, because of the local nature of the segmentation process, DL-based auto-segmentation is based on fully convolutional neural networks. The architecture of the adopted networks is mostly undisclosed, but some are reported to be based on modifications of the U-net. Examples^5–7^ are listed in the following:

- MIM Contour ProtégéAI based on U-net architectures,^8^ compared to atlas-based segmentation (MIM Maestro)^9^;
- Mirada Medical DLCExpert based on multiple U-nets, compared to atlas-based segmentation (ABAS software, Mirada Medical)^10^;
- Therapanacea ART-Plan Annotate based on an ensemble of DL models^11^;
- RaySearch Laboratories, RayStation v9B and v10A based on fully convolutional neural networks^12^ and RayStation 2023B optimized for scanned proton beams^13^;
- AI-Rad Companion Organs RT, Siemens Healthineers^14^;
- ADMIREv.3.41, Elekta AB, based on U-net architecture.^11^

#### Biology-driven target identification

Biology-driven target identification refers to the exploitation of functional information offered by molecular imaging to improve the definition of the target. In particular, involved nodal radiation therapy (INRT) relies on the inclusion of malignant lymph nodes (LNs) in the radiotherapy target. LN invasion is typically assessed by means of fluorodeoxyglucose-based PET (FDG-PET), which has known spatial resolution limits, and surgical staging. Surgical staging is however an invasive procedure that can lead to postsurgery morbidity and delays in therapy start. AI models able to noninvasively and accurately assess LN invasion could play a role in extending the INRT application and improve radiotherapy outcomes.

Sher et al were the first to investigate an AI-based INRT framework inside a phase II clinical trial for head and neck (HN) squamous cell carcinoma (HNSCC) (INRT-AIR, https://clinicaltrials.gov/study/NCT03953976).^15^ HNSCC definitive radiotherapy ordinarily includes elective neck irradiation. Chen and colleagues developed an LN classification model based on both hand-crafted and DL-derived features, working on FDG-PET and contrast-enhanced CT images.^16^ In the clinical trial, 67 patients were enrolled and only LNs classified as malignant by the AI model were included in the target. Excellent oncologic outcomes and patient-reported quality of life were observed, supporting additional prospective studies that, if positive, could lead to an implementation of the model in the clinical settings.^15^

Lucia et al developed and assessed, on a multicentre dataset, 2 neural network models to predict para-aortic LN involvement in locally advanced cervical cancer. The first model takes as inputs 2 hand-crafted radiomic PET features (a texture feature and a morphological feature) computed inside the primary tumour volume and properly harmonized among different centres, relying on the removal of nonbiological sources of variance in imaging biomarkers in multicentre studies (ie, ComBat). The second model uses the clinical standardized staging from the International Federation of Gynecology and Obstetrics (ie, the FIGO stage 2018) as the third input. On the 3 test sets, both models achieved an area under the receiver operating characteristic curve (AUC) larger than 0.9, to be compared with an AUC ranging from 0.62 to 0.69 for a clinical model relying on FIGO stage 2018, tumour size and pelvic LNs on PET/CT.^17^ These promising models have not been assessed yet inside an INRT clinical trial but offer evidence of the emerging role of AI in providing these decision-making tools.

### Biology-driven radiotherapy dosing

In current clinical practice, treatment planning aims at matching dose-volume requirements and constraints in targets and OARs, respectively. To this aim, standardized dose/fraction regimens are used. Analytical population-based radiobiological tumour control probability (TCP) and normal tissue complication probability (NTCP) models cannot be used to personalize treatment. Radiosensitivity in targets and OARs varies indeed among patients, tumour, and organ characteristics, which these models do not consider. AI is expected to allow the construction of comprehensive data-driven outcome and toxicity prediction models that, properly associated with optimal decision-makers, could be used to personalize dose/fraction regimens in both target and OARs.^18^

Lou and colleagues in 2019 proposed a baseline (ie, pretreatment) DL framework predicting local failure and calculating a personalized optimal dose for lung cancers treated with stereotactic radiation therapy (SRT). The framework is composed of (1) Deep Profiler, a DL block taking in input pretreatment CT and gross tumour volume and generating an image-based failure risk score; (2) iGray, a multivariable regression model relying on image-based failure risk score, biologically effective dose and histological subtype, able to predict local failure and to calculate a personalized dose that ensures a treatment failure probability <5% at 24 months. The DL framework, trained on 849 patients, most receiving 50 Gy in 5 fractions, was assessed on an independent 95 patient test set, where it predicted treatment failure with an AUC of 0.77.^19^ The Deep Profiler + iGray framework is currently under assessment in a prospective clinical trial (RAD-AI, https://clinicaltrials.gov/study/NCT05802186), in which the personalized dose is applied and local failures are evaluated.

Other works in the literature exploit AI to create baseline radiotherapy outcome models, including carbon ion beam therapy.^20^^,^^21^ However, these models have not yet been associated with optimal decision-makers, nor inserted into any dose/fraction regimen personalization workflow.

As to tumour control modelling, Vallières et al in 2017 were among the first to propose and assess on an independent cohort, an HN tumour failure model relying on hand-crafted radiomics features computed on PET-CT pretreatment images.^22^ Regarding instead the use of DL radiomics features, Jalalifar et al proposed a DL framework to predict local failure in brain metastases treated with SRT. The framework relies on treatment planning contrast-enhanced T1 and T2-FLAIR MRI and on clinical parameters (histology, tumour location and size, number of brain metastasis). It consists of a 2D convolutional neural network (CNN) (InceptionResNetV2) that extracts features from 2D images in treatment planning structures including oedema and of a recurrent network (ie, a long short-term memory network) that takes as inputs imaging features along with clinical parameters and incorporates spatial dependencies between 2D images. The model, trained and optimized on 156 lesions, obtained an accuracy of 82.5% when tested on an independent dataset of 40 lesions, thereby showing very promising results when compared to an accuracy of 67.5% achievable with clinical features only. Heat maps show that lesion margin areas are those mainly influencing the predicted outcome.^23^

As to toxicity modelling, several authors proposed to overcome the limits of conventional NTCP models based on dose-volume histogram (DVH) features by extracting radiomics features from 3D dose maps (dosiomics). Bourbonne et al proposed hand-crafted dosiomics toxicity models for lung cancers treated with volumetric modulated arc therapy. The model has been trained on 117 patients for acute and late lung toxicity prediction. On the 50 patients’ independent test set, the proposed models obtained a balanced accuracy (BAcc) of 0.92 for acute lung toxicity and 0.89 for late pulmonary toxicity, while models relying on clinical/DVH features obtained a BAcc of 0.69 and 0.80, respectively.^24^ Again, in lung cancer intensity modulated radiotherapy, Lee et al and Zheng et al proposed to combine hand-crafted dosiomics features with hand-crafted radiomics CT features to, respectively, predict acute phase weight loss and acute radiation esophagitis.^25^^,^^26^ Men et al proposed to combine dose maps and CT information into a 3D residual CNN model to predict xerostomia in HNSCC. The model inputs are planning CT images, dose maps, parotid and submandibular gland contours. On a test set of 78 patients, an AUC of 0.84 was obtained.^27^ Cui et al proposed a multiomics model for NSCLC able to simultaneously predict time-to-event probabilities for local control and radiation pneumonitis. The model relies on dose parameters, PET hand-crafted radiomics features as well as biological information (ie, cytokines and microRNAs). It consists of variational autoencoders for feature selection, NNs, and survival neural networks. The model has been assessed on both internal and external test sets, providing an AUC of 0.70 for radiation pneumonitis and 0.73 for local control.^28^ Wei et al recently proposed a DL model for the prediction of liver toxicity in stereotactic body radiation therapy (SBRT) of hepatocellular carcinoma (HCC), that relies on pretreatment MR hepatobiliary contrast uptake rate maps and treatment dose maps. Post-treatment contrast uptake rate maps are estimated with a conditional GAN with Waserstein loss; NTCP is modelled starting from estimated pre-/post-treatment contrast uptake rate change and treatment dose maps. On a small patient cohort, the model has shown promising albeit preliminary results.^29^

### Treatment plan calculation

#### Anatomy-based auto-planning

Conventional treatment planning requires inverse optimization to determine the radiation beam parameters that match the prescribed dosimetric criteria for controlling the tumour, including constraints accounting for the radiosensitivity of OARs and normal tissue. These criteria are expressed as dose and volume scalars (eg, homogeneity index), DVHs, or even as a reference dose distribution. The optimized parameters can be manually adjusted with time-consuming and labour-intensive trial-and-error workflow, especially in highly conformal treatment modalities. To automatize the exploration of the trade-off between multiple dosimetric criteria, multi-criteria optimization has been introduced to support the selection of the treatment plan. Therefore, conventional treatment planning is a computer-aided but ultimately human-driven process.

The automation of treatment planning is fundamentally based on the anatomy-to-dose correlations inferred from a cohort of clinical treatment plans. This is generally referred to as knowledge-based radiation therapy treatment.^30^ Automatic prediction of the dose distribution can be based on atlas that are adapted to the patient according to optimization algorithms, including DIR.^31^ Dose mimicking optimization then converts the reference dose distribution to a deliverable treatment plan. AI methodologies, with particular reference to DL and machine learning (ML), have been recently proposed to automate different stages of the workflow for improving treatment planning quality and efficiency, including the selection of beam angles.^32^ AI-based auto-planning refers to the prediction of the DVH and the dose distribution.^33^^,^^34^ DL is also reported to estimate the radiation beam parameters without inverse optimization.^35–38^

The AI-based prediction of the dose distribution has been typically based on fully convolutional neural networks combined with residual connections such as Res-Net,^33^ DoseNet,^39^ and modified U-net.^40^ The GAN architecture has been proposed to replicate the role of the treatment planner (ie, generator) and the role of the radiation oncologist that evaluates the treatment planner (ie, discriminator).^41^^,^^42^ Reinforcement learning has been also presented as the architecture to reward the treatment planning quality.^43^ The vision of a learning loop framework based on quantitative evaluation of treatment outcomes has been also put forward to virtuously integrate AI with human knowledge.^44^

Commercial knowledge-based radiation therapy treatment planning software includes the widely investigated RapidPlan in Varian Eclipse planning system (Varian Medical Systems, Palo Alto, CA, United States)^30^ and feature also solutions for adaptive radiation therapy (ART) workflow, such as Varian Ethos adaptive treatment planning system (Varian Medical Systems, Palo Alto, CA, United States)^45^ and RayStation v9B (RaySearch Laboratories).^12^

## Anatomy-based treatment verification and adaptation

To optimize the therapeutic outcome, radiotherapy is typically administered in a fractionated treatment course entailing a few days (for hypofractionated treatment regiments) up to several weeks (for standard fractionation) of almost daily dose applications. Hence, with the advent of more advanced beam delivery technologies, there is a more compelling need to verify that the daily patient anatomy reflects the initial patient model made at the early time of treatment planning (ie, treatment verification), and adapt the treatment plan in case large anatomical changes have occurred (ie, treatment adaptation). Moreover, in the case of moving targets, advanced technologies and methodologies are used to monitor the tumour motion (ie, tumour tracking and motion compensation) and account for motion in the treatment delivery.

### Pretreatment verification

When in-room volumetric imaging such as cone beam CT (CBCT) or MRI is available, the patient model can be updated based on the imaging acquired in the treatment room prior to radiotherapy. Hence, treatments not subject to intra-fractional motion (ie, static treatments) can be re-planned without additional re-scanning of the treatment planning CT image according to the online or offline ART workflow (Figure 1). The role of AI is relevant to the definition of models for converting the in-room imaging into a suitable image for treatment planning while accounting for the anatomical changes. Relying on periodic in-room imaging, this fundamental role can be extended to the definition of models that anticipate such anatomical changes. The predicted anatomical changes can be then accounted for in subsequent fractions relying on recurrent neural networks.^46^ The timing of the prediction can be even pushed forward at the beginning of the treatment relying on convolutional long short-term memory networks.^47^

#### CBCT imaging

CBCT is currently used for patient position verification. Treatment re-planning based on CBCT imaging requires Hounsfield Unit (HU) correction techniques for scattering and noise. Alternatively, the image quality of the treatment planning CT image is mapped onto the anatomy of the CBCT image relying on DIR,^48^ along with contour propagation. DL has been proposed for CBCT correction to enable treatment re-planning, according to the so-called synthetic CT image. For instance, Therapanacea AdaptBox (https://www.therapanacea.eu/our-products/) is a commercial software for DL-based CBCT correction for adaptive photon therapy.

DL-based CBCT corrections are either applied to CBCT in the projection domain prior to tomographic image reconstruction or directly on the reconstructed CBCT image. In the projection domain, the target of the training is represented by scatter-free and noise-free CBCT projections, either corrected or obtained relying on forward-projection of the CT images,^49^^,^^50^ but also generated using Monte Carlo simulations.^51^

In the image domain, the target of the training is represented either by the CT image^52^ or by the corrected CBCT image.^53^ Most of the works have applied CBCT correction in the image domain, typically based on the U-net configurations, but GAN architectures have been also proposed.^54^^,^^55^ Investigation of DL-based CBCT corrections for treatment planning has been reported in the literature for different anatomical regions (ie, HN, lung, prostate, and pelvis).

Because of the need for imaging dose minimization and/or acquisition time/space constraints, image reconstruction based on sparse in-room projections is intended to infer the volumetric 3D image relying on a population-based model. Domain conversion typically requires fully connected layers, sparsely connected if accounting for geometrical correspondence between image and projection domains. Inspired by sparse view CT image reconstruction,^56^ a recent trend suggests compressed sensing^57^ and dictionary learning^58^ based on a sparse representation of the image relying either on morphological image transformation according to Wavelet and Shearlet basis functions (ie, compressed sensing) or on prototype images (ie, dictionary learning). These representations enable a reduction of the size of the feature maps, similar to pooling operations in the encoding path of the network. Alternatively, domain conversion can be explicitly implemented in physics-informed networks, as unrolled algorithms for tomographic image reconstruction.^59^ DL-based CBCT correction during tomographic image reconstruction from projections, relying on domain conversion,^60^ is however not yet proposed for applications in radiotherapy.

#### MRI

The potential role of MRI in radiotherapy covers the entire ART workflow, spanning from treatment planning and re-planning, up to motion management and long-term treatment verification. The nonionizing properties and better soft tissue contrast of MRI are exploited to substitute X-ray imaging within the entire ART workflow. However, the major limitation of MRI in ART is the missing measurement of the electron density tissue properties, related to the X-ray attenuation coefficients expressed in HUs, and to the stopping power in ion beam therapy.

The conversion of an MRI image into a pseudo-CT image is introduced to overcome this limitation. To this end, the MRI is calibrated to a pseudo-CT image relying on DIR of the MRI atlas or based on DL.^61^ The training of DL-based MRI into pseudo-CT conversion is typically based on co-registered or “paired” MRI-CT images, relying on DIR. The registration profoundly influences the conversion accuracy.

A modified conditional GAN architecture has been proposed to account for potential registration inaccuracies. The mutual information between the synthetic CT image and the CT image has been used as the metric for the generator’s loss function to train non-aligned MRI-CT images.^62^ The conditional GAN architecture has been adopted in proton therapy^62^^,^^63^ and in rare studies about anatomically complex treatment sites (ie, abdomen) in carbon ion beam therapy.^64^ The need for “paired” MRI-CT images has been overcome by the cycleGAN architecture, which has been reported for proton beam therapy of liver^61^ and prostate.^65^ However, the U-net has been the typical architecture used for DL-based MRI into pseudo-CT conversion. Improved training of the U-net based on multiplanar image slices has been proposed for application in proton therapy,^66^ aiming at solving the problem of the low MRI signal of the skull bone that causes HU overestimation,^67^ along with the air cavity interface and thermoplastic mask.^68^ For this purpose, the use of the cycleGAN architecture has been also proposed.^69^

DL-based MRI conversion into pseudo-CT image is mostly investigated for treatment planning rather than ART due to the current limited availability of in-room MRI, for which expensive and not yet too widely spread instrumentation is available in photon therapy, and for which solutions for ion beam therapy are still under investigation due to the larger technical complexity and related costs. In the latter case of ion beam therapy, stricter accuracy requirements in the MR-based pseudo-CT generation are also needed because of the most demanding subsequent conversion to stopping power.

### Tumour tracking and motion compensation

When the patient model accounts for moving targets, intra-fractional motion monitoring systems including imaging systems are employed for tumour tracking and motion compensation in dynamic treatment sites.^70^ When the tumour is not directly visible in the in-room imaging, tumour tracking makes use of surrogates that correlate with tumour motion. Prediction models define the relationship between these surrogates and the tumour position at each motion state. Surrogates can be either external (ie, acquired by optical tracking systems as used in conventional internal-external prediction models) or internal (ie, acquired by in-room imaging systems as navigator images). The definition of subject-specific or population-based motion models, typically constructed as a prior relying on DIR between 4D images, enables the estimation of the anatomical motion state in terms of the volumetric 3D image for time-resolved dose calculation. Models that directly correlate the surrogates to the anatomical motion state^71^ or the volumetric 3D image^72^ are also proposed, thus paving the way toward ML-based motion modelling and motion compensation.

Despite the well-established use of neural networks for tumour tracking,^70^ ML-based motion modelling (ie, the inference of the deformation fields) and motion compensation (ie, the prediction of the volumetric 3D image) are emerging, particularly for in-room MRI applications. A population-based motion model has been inferred from 4D images by a neural network proposed by Romaguera and colleagues.^72^ In this work, patient-specific motion compensation is based on a multibranch (ie, 3 branches) CNN. The first branch is the motion encoder, to be applied to each motion state. This encoder maps the deformation field onto a low-dimensional space containing compact representations of the motion state. A second branch is an auxiliary encoder, dedicated to anatomical feature extraction from the patient-specific treatment planning 3D image. A third branch is the temporal predictive network, intended for delay compensation and thus, real-time motion compensation at the temporal resolution of the surrogate images.^73^ The compact representation of the motion states, linked to those of the patient-specific internal surrogates (ie, the cine MR images) and the treatment planning 3D image, is then fed into the decoder to predict the deformation field for the motion states of the surrogates, as a conditional variational autoencoder.

Relying on patient-specific models, the volumetric 3D image can be derived directly from in-room 2D projections to potentially enable ART in stationary irradiations, as well as real-time tumour tracking in dynamic treatment sites, along with the verification of internal-external prediction models.^74^ This “reconstruction” is obtained with a hierarchical neural network in an encoder-decoder framework. An encoder represents the 2D projections into a feature space. The learned features are then used to generate the 3D CT image by the decoder. The network decodes the hidden information in the 2D projection and predicts the volumetric 3D image based on prior knowledge gained during training, which is based on augmented 2D-3D data pairs of different body positioning and anatomical configuration.

To the best of the authors’ knowledge, there is no record yet of commercial approval of patient-specific models for ART applications (including tumour tracking and motion compensation).

### Transmission/emission-based treatment verification

Although the availability of an updated patient model in combination with the records of the beam delivery can already enable a calculation of the delivered dose, there are ongoing efforts in photon and ion beam therapy to also enable an online, ideally real-time verification of the delivered treatment. This can be achieved by measuring the transmitted X-ray radiation (photon therapy) or secondary emissions induced by the interaction of the therapeutic beam with the patient tissue (ion beam therapy).

#### Photon therapy: electronic portal imaging device imaging

In photon therapy, the photon intensity transmitted through the patient during the treatment delivery can be acquired by electronic portal imaging devices (EPID). EPID images are mostly used for geometrical patient positioning as rigidly linked to modern linac accelerators. However, EPID-based measurements have been also proposed for treatment verification (ie, dosimetry), according to both planar and volumetric approaches. The treatment verification entails the comparison of the EPID image with a prediction image based on the treatment planning X-ray CT image or an in-room volumetric image, even acquired with the EPID detector itself (ie, Mega voltage CBCT). DL has been adopted for EPID image correction for photon attenuation and scattering as well as for the identification of treatment inconsistencies such as anatomical changes, positioning inaccuracies, and mechanical errors.^75^ Recently, DL has also been proposed to predict 3D dose distributions inside the patients from EPID images, based on unsupervised learning (ie, GAN architecture^76^) as well as supervised learning relying on highly accurate Monte Carlo simulations (ie, U-net architecture^77^).

#### Ion beam therapy: imaging of secondaries

In ion beam therapy, no penetrating radiation is transmitted after the stopping of the beam in the tumour, but secondary physical emissions of a different nature are produced and can be detected outside the patient, which is a vivid subject of ongoing research and development. As an alternative to the indirect comparison of the measurement of these secondary emissions with a prediction based on the initial patient model and treatment plan, the actually delivered dose distribution can be retrieved from the distribution of the secondary emissions by means of “dose reconstruction algorithms.” DL has been proposed for dose reconstruction in the context of PET^78–80^ (ie, relying on the detection of annihilation photons produced by fragmentation of the tissue [and beam] nuclei) and prompt gamma imaging^81^ (ie, relying on the detection of energetic gamma rays produced in the fast de-excitation of nuclei after initial excitation through nuclear interaction between the beam itself and the tissue nuclei). Some of these approaches have been preliminarily investigated relying on the ground truth distribution of the secondary emission as obtained from computational models like Monte Carlo simulations, not accounting for the effects coming from the detection and the reconstruction of the events. With respect to that, DL has been proposed to close this gap and thus also retrieve the prompt gamma emission distribution based on the measurements.^82^

## Biology-driven treatment adaptation

AI is expected to provide tools not only for treatment personalization in the planning phase but also for personalized treatment adaptation. Several authors have shown that outcome models relying on parameters acquired both at baseline and during treatment perform better than baseline-only models.^83^ A personalized dose-escalation strategy can be, for example, a re-planning guided by tumour FDG-avid region shrinkage.^84^ The originally planned dose/fraction regimen can be however potentially adapted during the radiotherapy course by exploiting variations in clinical, biological, and radiomics parameters. In personalized biology-based treatment adaptation, AI is required (1) to identify prognostic baseline along with delta radiomics features and to combine them with clinical, dosimetric, and biological features into data-driven outcome and toxicity models, and (2) to exploit outcome and toxicity predictions during the treatment course to optimally adapt the treatment plan.

Methodologies for reproducible delta radiomics feature selection have been proposed in the literature.^85^ A comprehensive general framework for AI-based personalized treatment adaptation has been defined by Tseng and colleagues in 2018.^86^ By assuming to have optimal TCP and NTCP models, adaptation can be implemented with linear or nonlinear feedback control systems, or with reinforcement learning algorithms that search over all the possible decisions and identify the best strategy to optimize the probability of a positive outcome.^86^^,^^87^ Niraula and colleagues recently implemented an AI-based multiomics interactive optimal decision-making software (ARCliDS) to guide personalized treatment adaptation. ARCliDS is composed of (1) ARTE, a Markov decision process modelled via supervised learning that, starting from pre- and during treatment parameters and planned dose, gives an estimate of TCP and NTCP; (2) ODM, a reinforcement learning optimal decision-maker, that recommends optimal daily dosage adjustment to maximize TCP and minimize NTCP. ARCliDS has been retrospectively trained and applied on an NSCLC adaptive radiotherapy dataset and on an HCC adaptive SBRT dataset. In the learning phase, 13 multiomics features were selected for both applications, partly baseline and partly delta features. In the operation phase, ARCliDS was found able to reproduce 36% of good clinical decisions and improve 74% of bad clinical decisions in NSCLC treatment and to reproduce 50% of good clinical decisions and improve 30% of bad clinical decisions in HCC treatment.^88^

## Discussions and conclusions

This review has addressed the main tasks of the radiotherapy framework (Figure 1). Treatment selection and planning/adaptation of combined drug-radiation treatments have not been covered in this review. Preliminary works have tried to exploit AI even in these fields. On soft tissue sarcomas, where treatment selection strongly depends on tumour grading, MRI hand-crafted and DL radiomics have been proposed as non-invasive grading tools to replace biopsy.^89^ ML decision-making tools have been also proposed in HCC, to provide treatment recommendations for tumours undergoing transarterial chemoembolization,^90^ and to properly select between photon and proton therapy to minimize liver toxicity.^91^ As to the planning/adaptation of combined drug-radiation treatments, AI-based strategies will certainly play a central role in the near future.^92^

Overall, it has been shown that AI tools are both rapidly emerging in anatomy-based radiotherapy tasks currently covered by conventional methodologies and also opening up innovative biology-driven task that conventional methodologies cannot manage. As a matter of fact, in applications whose results are easily verifiable and correctable by expert operators (ie, auto-segmentation and auto-planning) AI tools have quickly entered the clinical practice in commercial solutions. Long-term verifiable applications, on the other hand, require extensive exploration before possible AI tools introduction into clinical routine.

Prior to the integration of radiomics (and dosiomics) into clinical practice for biology-driven tasks, it is imperative to address the acknowledged limitations of radiomics, an area where the scientific community is actively engaging.^93–95^ Upon overcoming these obstacles, AI is ready to revolutionize radiotherapy by offering a clear pathway towards a comprehensive personalization.

Regardless of the scope, the ability to select relevant features for prediction is at the heart of AI’s potential. With reference to this aspect, the importance of interpretability and explainability of the features themselves and of their role in the prediction represents the next commitment that the multidisciplinary community of scientists must face.^96^^,^^97^ Interpretability is concerned with “understanding the prediction,” explainability with “understanding the path that takes to prediction.” Interpretability and explainability are fundamental elements to handle the ethical implications of AI in radiotherapy.^98^ Although the interpretability for most of the AI methodologies has been significantly developed, full explainability has not yet been achieved. In particular, there is generally a trade-off between accuracy and interpretability, that is the interpretability potential worsens with prediction accuracy improvement.^99^ Another important element regarding the reliability of the prediction, mostly concerned with the “understanding of what is not actually predicted,” is the quantification of model uncertainties (ie, epistemic uncertainties) and the stochastic uncertainties.^100^ To this purpose, the synergistic interaction between human knowledge, including the knowledge embedded in conventional methodologies (Table 3), and AI is envisioned as the way towards reliable AI applications in radiotherapy.^101^

**Table 3.** The role of AI, including AI-based methodologies, for the different tasks within the personalized radiotherapy framework.

		Conventional methodology	AI-based methodology			
			Tasks	Role of conventional methodology	Methodology	Clinically used
Biology-driven workflow	Personalized radiation treatment	—	Definition of data-driven models based on large amounts of data; patient outcome improvement	—	ML (ie, multivariate models for classification and regression) and DL	No
Anatomy-based workflow	Auto-segmentation	Manual segmentation	Improvement of the workflow (automation, time reduction) and improvement of the task (variability reduction)	For AI training	DL (ie, U-nets and modified U-nets)	yes
	Auto-planning	Computer-aided but human-driven treatment planning	Improvement of the workflow (automation, time reduction) and improvement of the task (variability reduction)	For AI training	ML and DL (ie, Res-Net, DoseNet, modified U-net, GAN, reinforcement learning, etc.)	yes
	Pretreatment verification	Computer-based and human-supervised deformation models	Improvement of the workflow (automation, time reduction) and improvement of the task (variability reduction)	Not used. The data-driven model overcomes the limitations of deformation models	DL (ie, modified U-net, GAN, cycleGAN, conditional GAN)	No
	Tumour tracking and motion compensation	Computer-based and human-supervised correlation and deformation models	Improvement of the workflow (automation, time reduction) and improvement of the task (variability reduction)	Not used. The data-driven model overcomes the limitations of correlation and deformation models	DL (ie, encoder-decoder architectures)	No
	Transmission/emission-based treatment verification	Computer-based	Improvement of the workflow (time reduction)	For AI training	DL (ie, U-net, modified U-net, GAN)	No

Abbreviations: AI = artificial intelligence; DL = deep learning; GAN = generative adversarial networks.

## Supplementary Material

tzae017_Supplementary_Data
