# Supplementary material for: “Under the hood”: artificial intelligence in personalized radiotherapy
Source: BJR Open. 2024 Jul 16;6(1):tzae017. doi: 10.1093/bjro/tzae017 (PMC11299549; doi:10.1093/bjro/tzae017)
Supplement: tzae017_Supplementary_Data [file tzae017_supplementary_data.pdf]

Appendix

Artificial neural networks and deep learning

Among machine learning (ML) approaches, artificial neural networks (ANN) rely on elements, neurons or nodes of the network, designed in analogy to the neuronal cell. The neuron consists of a group of input signals that undergo a linear operation (i.e., weighted sum of the input signals plus a bias) simulating synaptic integration and subsequently a non-linear operation or activation function (i.e., a rectified linear unit or “ReLU”) simulating the action potential, thus generating an output signal. The output of a neuron is fed to another neuron as one of the inputs. The widely used convolutional neural network (CNN) are designed to simulate the visual nervous system so that the linear operation of the neuron becomes image convolution or actually cross-correlation. The cross-correlation is a weighted sum over a local region of the image, described as a kernel, so that the neuron of a layer is connected only to this region of the preceding layer, thus reducing the number of parameters of the network. For instance, the kernel represents the receptor whereas the local region represents the receptive field. The neurons of the CNN are organized according to the spatial dimensionality of the image (i.e., height, width for two-dimensional image) and to the number of kernels (i.e., depth or channel dimension). Deep learning (DL) refers indeed to these characteristics of the CNN. The kernels are applied to the input image to generate multiple output channels. The channels are intended to describe different aspects (feature maps) of the image. The generalization of this multi-dimensional array is referred to as tensors. Pooling layers (i.e., maximum pooling, average pooling...) sub-sample the image to encode features of the input image into a latent space of a relatively lower dimension, thus introducing feature levels to the network and reducing the number of parameters. The decoding of these features requires un-pooling layers or up-convolutional layers. Deep networks are typically provided with encoding and decoding paths, eventually combined with skip (i.e., concatenation of the tensors) or residual (i.e., summation of the tensors, so that the skipped layers learn the “residue”) connections to feed the output of the contracting/down-sampling layers as the input to the symmetrically correspondent expanding/up-sampling layer. Skip and residual connections are introduced to avoid vanishing gradient in back-propagation and reminding image details to the expanding layers, as the well-known U-net originally proposed for semantic segmentation. Complex architectures can be derived to explicitly model the relationship between inputs and outputs of two or more deep networks as in generative adversarial networks (GANs, conditional GAN, cycleGAN...).

Radiomics and dosiomics

Radiomics is concerned with the extraction and analysis of quantitative, and ideally reproducible, information from medical images. Radiomic features extracted from X-ray CT images, as well as from PET images and MRI, can be used to predict clinical end points in patients undergoing radiation therapy, typically in terms of tumor control probability and radiation-induced toxicity. Dosiomics extends the concept of radiomics to the dose distribution of the treatment planning. The underlying assumption of radiomics and dosiomics is that these imaging/dose features describe diagnostic and prognostic

characteristics of tumors and surrounding organs. Radiomics is coupled to ML to select end-point related, non-redundant biomarkers inside the large amount of computable hand-crafted features. DL instead directly handles the images to automatically extract inherent or deep radiomic features. ML classifiers and regressors, typically trained with supervised learning, can be then used to construct multivariate models relying on imaging, clinical, histologic and genomic biomarkers, that can be putatively used for treatment personalization.
